# Life situation of women impaired by Thalidomide embryopathy in North Rhine-Westphalia - a comparative analysis of a recent cross-sectional study with earlier data

**DOI:** 10.1186/s12905-019-0745-y

**Published:** 2019-04-03

**Authors:** Christina Samel, Christian Albus, Irmgard Nippert, Alexander Niecke, Markus Lüngen, Holger Pfaff, Klaus M. Peters

**Affiliations:** 1 Institute of Medical Statistics and Computational Biology (IMSB), Faculty of Medicine, University of Cologne, Bachemer Str. 86, 50931 Cologne, Germany; 2 Institute for Health Economics and Clinical Epidemiology, University of Cologne, Gleueler Str. 176-178, 50935 Cologne, Germany; 3 Department of Psychosomatics and Psychotherapy, University Hospital of Cologne, Kerpener Str. 62, 50937 Cologne, Germany; 4 Institute of Human Genetics, Westfälische Wilhelms-Universität Münster, Vesaliusweg 12-14, 48149 Münster, Germany; 5 Faculty of Business Management and Social Sciences, Osnabrück, University of Applied Sciences, Postfach 19 40, 49009 Osnabrück, Germany; 6 University of Cologne, Faculty of Human Sciences and Faculty of Medicine, Institute of Medical Sociology, Health Services Research, and Rehabilitation Science (IMVR), Eupener Str. 129, 50933 Cologne, Germany; 7 Department of Orthopedics and Osteology, Dr. Becker Rhein-Sieg-Klinik, Höhenstr. 30, 51588 Nümbrecht, Germany

**Keywords:** Thalidomide, Health outcomes, Birth defects, Congenital disorder, Embryopathy, Dysmelia, Healthcare, Unmet medical needs, Follow up study

## Abstract

**Background:**

Between 1957 and 1961 the substance Thalidomide was sold in West Germany and taken by many women as a sedative during pregnancy. This lead to miscarriages and infants been born with several severe malformations. The aim of this study was to describe the current situation of women impaired by Thalidomide induced embryopahty in North Rhine-Westphalia (Nordrhein-Westfalen), Germany, in comparison with the results found in a study done in 2002 by Nippert et al.

**Methods:**

Questionnaires as well as examinations were performed. Data were compared using descriptive and inductive statistical methods.

**Results:**

Both studies show that women impaired by Thalidomide embryopathy face a poorer health status than women their age in the general population and live in fear of further deteriorating health. The majority can only work reduced hours or are already retired due to poor health. Most of those who need assistance are being assisted by their social environment, while professional care is still utilized in only few cases.

**Conclusions:**

An obvious need for a shift in the provision of assistance and/or care provided was found as the social environment supporting the impaired women is also aging and therefore in high danger of breaking apart.

**Trial registration:**

The study has been registered at German Clinical Trials Register, DRKS00010593, on 07.06.2016 retrospectively.

## Background

Between 1957 and 1961 the substance Thalidomide was sold in West Germany under the trade name “Contergan”. The drug was sold in many other countries like Sweden, Brazil, Ireland, the United Kingdom, Japan, Australia and Canada under several names [1, 2]. It was explicitly advertised in Germany by the manufacturer among physicians and pharmacists as a sedative being safer than other drugs [3, 4], and in other countries as “free from untoward side effects” [5]. Furthermore the impression was left deliberately that it could be used safely by pregnant women [6, 7], so many of them took it during pregnancy. But, while being taken in the first trimester of pregnancy, Thalidomide can cause severe fetal damages [8]. The incidence of miscarriages and perinatal morbidity remains unknown with widely varying estimations [1]. Almost 90% of the children born in Germany with impairments caused by Thalidomide show dysmelias, either in their upper extremities alone or in their upper and lower extremities. Further disfiguring impairments in the face and hearing loss could occur [9]. A population study done in Sweden showed an elevated risk for autism in combination with mental retardation [10].

Two independently working clinicians expressed concerns about a connection of the drug with the witnessed rise in the incidence of those very specific birth defects [2, 11, 12]. In consequence this led to the withdrawal of Thalidomide from the German pharmaceutical market on November 26, 1961. In Canada the authorities took action on March 2, 1962 [13], while in Japan some of the manufacturers withdraw Thalidomide voluntarily on May 17, 1962 [14] from the market.

While the exact causation mechanisms for the birth defects are still not fully understood and many theories are presented [7, 15–17], the issue of impairments generated by Thalidomide has not lost any of its relevance. In recent years the agent has come back into the drug market for treatment of leprosy [18], cancer [19–21] and refractory Crohn’s Disease [22]. Although being distributed under strict regulations [23–25] in some countries children affected by Thalidomide have been born [18, 26, 27] due to the renewed use of the drug.

By now, 55 years after withdrawal of the drug from the pharmaceutical market, the individuals suffering impairments from the substance face subsequent damages of their disablement, which often include chronic pain. Those subsequent damages have an enormous impact on the impaired persons’ way of life, as they cause long sick leaves, dependency on wheelchair and possible resulting social isolation with all its consequences [28, 29].

In 2002 Nippert et al. [30] (“N2002”) conducted a survey among women affected by Thalidomide from North Rhine-Westphalia to gain better understanding of long-term consequences of the impairments. In this survey it was found that those women judged their health-related quality of life and their health care poorer than women their age from the general population living in the same area. In 2015 a study financed by the NRW Centre for Health (Landeszentrum Gesundheit Nordrhein-Westfalen) was presented by Peters et al. [29] (“P2015”) which aims were to systematically identify and document the congenital and subsequent impairments caused by Thalidomide.

No longitudinal study that the authors know of has been done to describe the ongoing deterioration of health in Thalidomide affected persons. The aim of this publication is to compare the previously collected data of N2002 with recently collected data of P2015 and therefore try to close this gap of knowledge as to current health problems and anticipated health problems, medical care and assistance.

## Methods

### Study design

Both studies were cross-sectional studies.

### Eligibility criteria and recruitment process

**Fig. 1:**
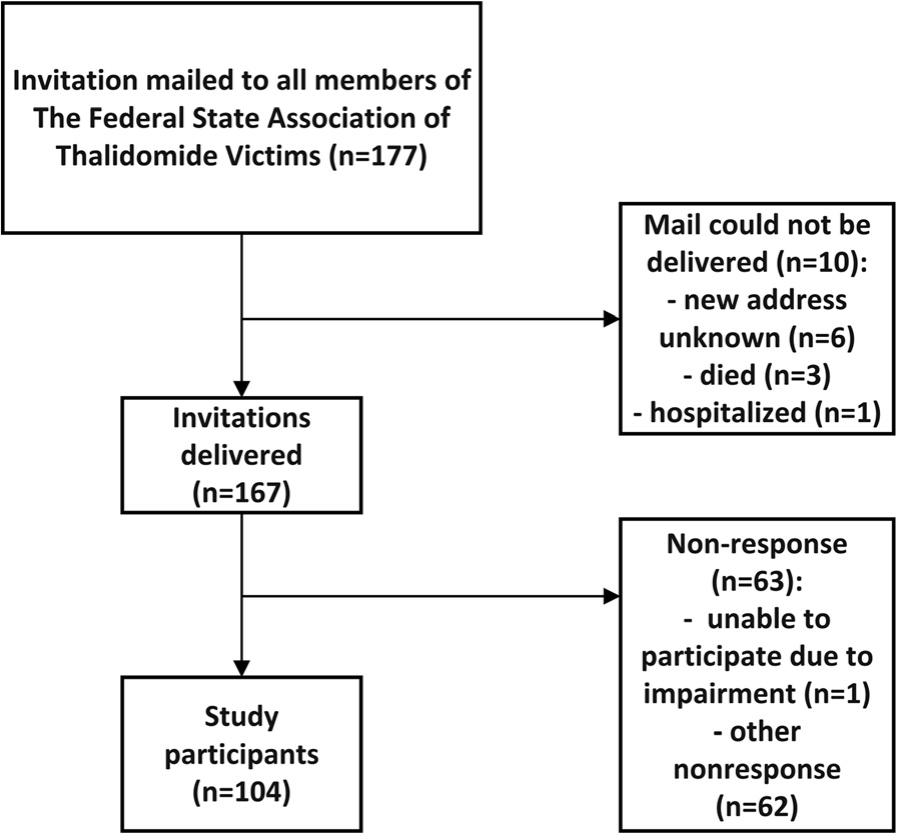
Flowchart recruitment process of N2002

In N2002 the women were recruited by the patient support organization The Federal state Association of Thalidomide victims (North Rhine-Westphalia) (Interessenverband Contergangeschädigter Nordrhein-Westfalen e. V.), which mailed all 177 of their female members in January 1999 and asked them to participate in the survey. Ten letters could not be delivered and one woman was unable to participate due to her impairment (see Fig. 1), while 104 women (62.6%) took part in the study.

**Fig. 2:**
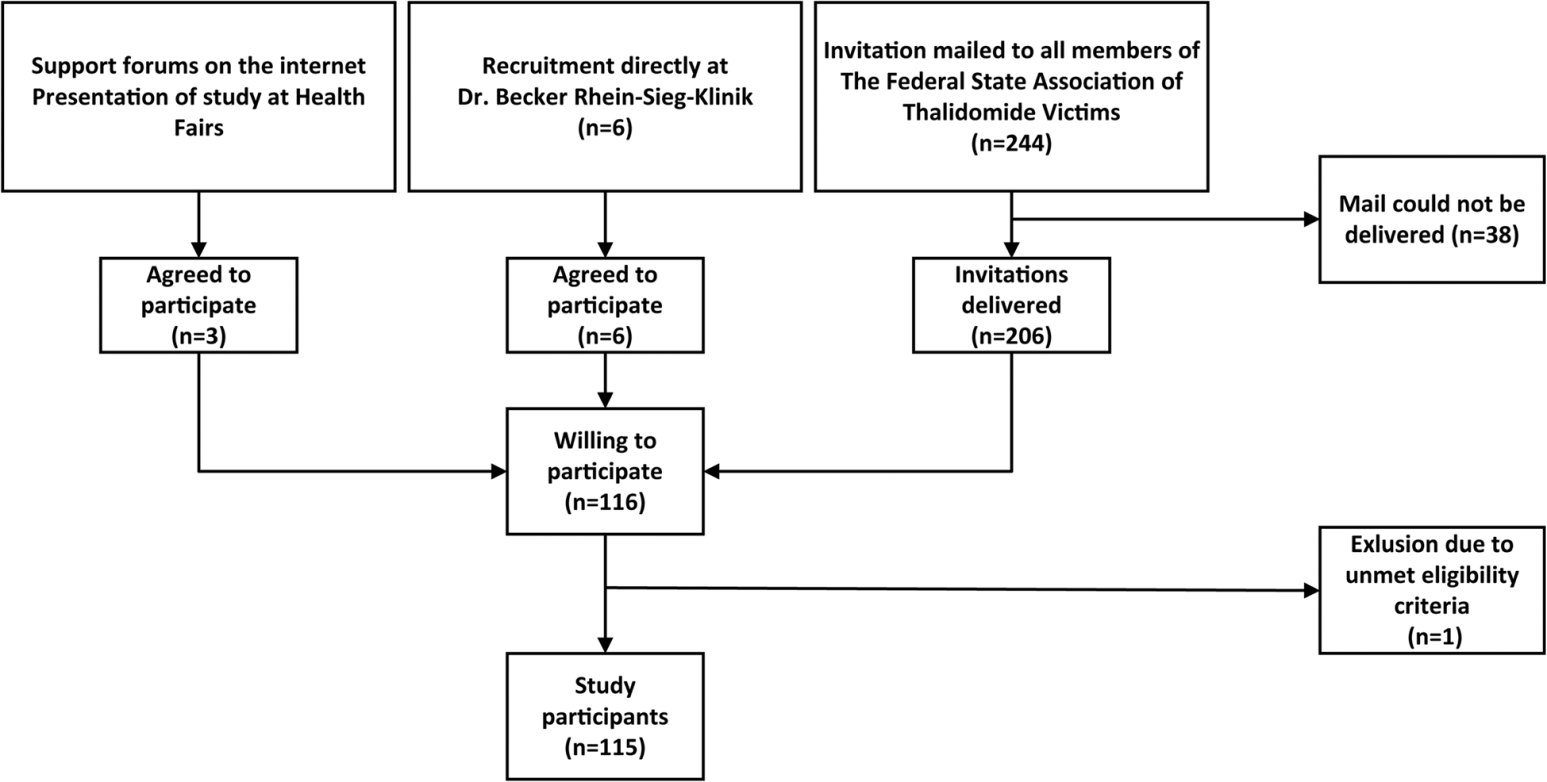
Flowchart recruitment process of P2015

In P2015 all females impaired by Thalidomide that were recognized as suffering from the effects of Thalidomide by the Contergan Foundation for Disabled People (Conterganstiftung für behinderte Menschen) were eligible if they were either currently living or were born in North Rhine-Westphalia, the most populated state in Germany. Since the study-team was denied access to the addresses of the women affected by Thalidomide by the Contergan Foundation for Disabled People due to data regulations, the participants were also recruited through the patient organization The Federal state Association of Thalidomide victims (North Rhine-Westphalia). The organization mailed out in September 2011 information sheets about the study to potential participants. Along was mailed the offer to be accompanied by a peer when taking part. The potential participants were also offered to get a full medical report of all diagnoses found separated by congenital and subsequent impairments as well as individually recommended treatment options after being examined. Of the 244 letters mailed, 38 letters could not be delivered and were returned. Two reminders were sent, one in May 2012 and one in January 2013. A total of 115 women responded and took part in the study which is a return rate 55.8% of those successfully contacted. It was attempted to reduce a possible selection bias coming from recruiting via a patient organization by advertising the study on the internet, on health fairs and by recruiting individuals that were known not to be organized in support groups directly at the Dr. Becker Rhein-Sieg-Klinik, Nümbrecht (see Fig. 2).

### Examinations

In N2002 no physical examinations and psychological evaluations were made. All data was collected by a questionnaire.

In P2015 all individuals were systematically physically examined by the same two orthopedists. Where needed, x-ray or sonography was performed. Damages found were categorized as congenital or subsequent disabilities. An evaluation of mental disorders was performed by either a trained and registered psychologist or psychiatrist using the German version of the structured clinical interview for DSM-IV [31], screening both SCID-I [32, 33] and SCID-II [34]. The SCID-I interview allows to diagnose for all major mental disorders while the SCID-II interview allows to diagnose personality disorders. The results found in the mental evaluation have been previously published [35]. In N2002 no mental evaluation was performed.

In P2015 the individuals had both the physical examination and psychiatric evaluation in one day. All examinations and evaluations took place between October 2011 and May 2013 at the Dr. Becker Rhein-Sieg-Klinik, Nümbrecht, Germany.

### Questionnaire

In N2002 a questionnaire was developed that contained questions about socioeconomic characteristics, current medical conditions, access to health professionals and medical care as well as the extent of assistance needed [30]. Quality of life was assessed using the German Version of the WHO QOL-BREF [36, 37].

In P2015 the questionnaire included questions on socio-economic and –demographic data, comorbidities, medical care, satisfaction with the medical care received and extent of care needed [38, 39]. For further assessment of health-related quality of life the German version of the EQ-5D descriptive system [40–42] was used. The questionnaires were sent to the participants after the written consent was given. The individuals were asked to bring the filled questionnaires to their appointment for the examination and psychological evaluation. Impaired individuals that were not able to fill in the questionnaire by themselves were assisted prior to the examinations in accordance to their needs. They were also given the possibility to be assisted by peers if desired.

The questions selected from the questionnaires used in both studies were tailored to identify and compare disease specific problems [30, 36–40, 42]. Net incomes were estimated for both studies by calculating the mean for grouped data. To compare the incomes between N2002 and P2015, the income of 2002 was converted to Euro with the exact exchange rate [43] and adjusted with the official German rate of inflation for each year [44] until 2014.

### Data analysis

The data was analyzed using IBM SPSS Version 21.0 and R Version 2.15.1. Counts and frequencies were used to describe the sample and contingency tables were calculated. For tests of differences in means two sample t-tests were performed, for tests of differences in proportions two-tailed exact binomial tests were used. To test for homogeneity, χ^2^-tests were used; to analyze 2 × 2 contingency tables exact Fisher tests were used. All reported *p*-values are two-sided. As this is a highly descriptive evaluation, all tests performed are of explorative nature to generate further hypotheses. Therefore no adjustment for multiple testing has been performed. Additional data that was collected in 2002 was provided by the author.

## Results

In this section the results are presented in comparison for N2002 and P2015.

### Background characteristics of the sample

Table 1 shows the socio-demographic and socio-economic differences between the populations in N2002 and P2015:

**Table 1:** Sociodemographic and -economic data of N2002 and P2015

	N2002, (*n* = 104)		P2015 (*n* = 115)		*p*-value
Mean Age (years)	38.0		50.5		not tested^+^
Educational background					< .001^*^
University degree (n,%)	19	18.3	41	35.7	
Baccalaureate/College (n,%)	26	25.0	19	16.5	
Secondary school (n,%)	40	38.4	45	39.1	
Less than secondary school (n,%)	19	18.3	10	8.7	
Occupation^a^					< .001^*^
Full-time (n,%)	38	36.5	10	8.7	
Part-time (n,%)	22	21.2	33	28.7	
Self-employed (n,%)	3	2.9	7	6.1	
Retired (n,%)	9	8.7	34	29.6	
Non-working/jobless (n,%)	24	23.1	9	7.8	
Other (n,%)	0	0.0	22	19.1	
Missing (n,%)	8	7.8	0	0.0	
Relationship status					not tested^+^
Single (n,%)	43	41.3	26	19.1	
Married/domestic partnership (n,%)	44	42.3	68	59.1	
Other (n,%)	17	16.3	21	18.3	
Having a child/children					not tested^+^
Yes (n,%)	38 ^c^	36.5	≥ 32^b^	≥ 27.8 ^b^	
No (n,%)	66	63.5	≤ 83 ^b^	≤ 72.2 ^b^	
Persons living in household					.891
One or tw (n,%)o	60	57.7	65	56.5	
Theree or more (n,%)	44	42.3	50	43.5	
Pension received by Conterganstiftung					.057
Yes (n,%)	98	94.2	111	96.5	
No (n,%)	6	5.8	1	0.9	
Missing (n,%)	0	0.0	3	2.6	
Estimated mean income (EUR)	1723.55		2036.48		not tested^+^

^*^tested significantly at .05 level

^+^tests not performed due to incomparability of instruments

^a^tested w/out other and missings

^b^asked only indirectly therefore underestimation is likely but represents a lower bound

^c^changed due to typing error in original publication, authorized by I. Nippert

The mean age in the sample of N2002 the mean age was 38.0 years with a range from 35 to 40 years, while in the sample of P2015 the mean age was 50.5 years (sd = .977 yrs) with a range from 48 to 53 years.

The participants in P2015 have reached significantly higher levels of education than the individuals in N2002.

The occupational situation differs significantly: many women were working full time in 2002 while in 2015 many of them worked only part time or have undergone early retirement due to poor health, see Table 1. Full time occupation was 36.5% in the N2002 sample and 8.7% in the P2015 sample. Part time working was increased in comparison and the number of individuals with a permanent legal disability to work seems to have risen in the past 12 years. The legal disability status for work is found to be significantly higher in P2015 (*p* < .001) than in the German female general population at that age, in which only 4.5% are legally disabled for work [45].

Of those who were still working in P2015, 38 (52.1%) women had an adapted workplace suitable for their disabilities. No sick leave in the last 12 month were reported by 14 (19.2%) women, short sick leaves up to 3 days were reported by 3 (4.1%) of the women. Longer periods of sick leave between 4 and 30 days were reported by 12 (16.4%), long term sick leave consisting of 31 or more days in the last 12 month were reported by 36 (49.3%) of the individuals. There were eight non-responders in this item.

Both studies investigated the relationship status but used different approaches. While in N2002 was asked for the marital status, in the inquiry done by P2015 asked for “living together/married”. Therefore, statistical tests could not be performed due to incomparableness of the items. Unfortunately, also the data concerning parenthood are not very well comparable since in P2015 this item was surveyed only indirectly through household situation. Only 27.8% of the Thalidomide-impaired women were living in 2014 with their child/children in a household. With this kind of question the proportion of women affected by Thalidomide having one or more children might be underestimated, but the proportion represents a lower bound. In contrast the survey N2002 asked directly for having a child/children and 36.5% stated they were a parent. As above, testing procedures were not performed. Both studies also show significantly lower proportions of Thalidomide-impaired women being a parent than the 78.6% of women in that age-group that have children in the general population in North Rhine-Westphalia [46], (N2002: *p* < .001, P2015: *p* < .001).

The number of persons living in a household was very similar in both study groups. In the N2002 study 24 (23.2%) of the women lived in a single-person household while in the P2015 sample 27 (23.5%) women lived on their own. While in N2002 the women affected by Thalidomide seemed to live in comparable household conditions as in P2015, the proportion of women living alone was higher in both studies than in the general population in North Rhine-Westphalia in the corresponding age group [47].

In N2002, the estimated mean net income was DM 3370.98 (€ 1723.55), which converts to an inflation-adjusted income of € 2072.47 in 2014. In the P2015 sample the estimated mean income was € 2036.48. The estimated household net income in both samples was around €1000 below the one in the general German population with € 3069 [48].

In N2002, 96.7% of those actively employed stressed the fact that their income secured their economic independence. While then 94.2% received a pension by Contergan Foundation for Disabled People (renamed from “Disabled Children’s Relief Foundation” (Hilfswerk für das behinderte Kind) in 2005 [49]), in P2015 of 111 women giving information about this topic all except one (99.1%) received a pension from this relief organization. The organization is fully funded by the state, therefore the compensation paid to the impaired is ultimately provided by the German government.

### Current health problems and anticipated health problems

In the P2015 sample 92 (80.0%) of the individuals were faced with impairments in their upper extremities and 11 (9.6%) in their upper as well as their lower extremities as congenital disabilities. A group of 12 (10.4%) individuals had no impairments in their limbs but are afflicted by other impairments caused by Thalidomide such as facial disfigurements (facial nerve paralysis, 11 cases (9.6%), (soft) palate paresis, 6 cases (5.2%)). N2002 did not collect this information.

In N2002 five women (4.8%) reported they had problems with their ears and eyes in the last 12 months. In the P2015 sample 33 (28.7%) women were diagnosed with congenital impairments in their eyes (*p* < .001), such as ophtalmoplegia in 29 cases (25.2%), lagophtalmos in 12 cases (10.4%) and blindness in one case (.9%). Among the 115 females 17 (14.8%) suffer from hearing loss (*p* = .023).

While in the N2002 sample 50.5% of the women affected by Thalidomide reported that they were “very satisfied” or “satisfied” with their health, in P2015 a significant lower proportion (*p* = .005) of 37.4% of the women reported that their health was “very good” or “good”. None of the women reported an “excellent” status of health. Their health-related status was described by 59.9% as “fair” or “poor”.

In N2002, 41.6% reported a decrease in health status over the last 12 months. In the P2015 sample 60.9% stated that their health-related status was “somewhat worse than 1 year ago” or “much worse than 1 year ago” (*p* = .040) while only 7.9% reported that their status of health had improved or stayed the same (27.0%). There were 5 (4.4%) non-responders to that question.

In N2002 64.4% did expect their health to further deteriorate in the next two years. In P2015 were 58.3% expecting that their health status will deteriorate further while only 13.8% did not expect a decrease in their status of health. This finding did not test significantly (*p* = .668).

Negative feelings such as blue mood, despair, anxiety or depression were never experienced by the individuals in 10.8% of the cases in N2002 and 75.7% stated that they were “very well” or “well” able to get around. 26.2% of the women reported that they felt pain was preventing them what need to do. Also did 68.0% report that they feel “very satisfied” or “satisfied” with their ability to perform their daily living activities.

**Fig. 3:**
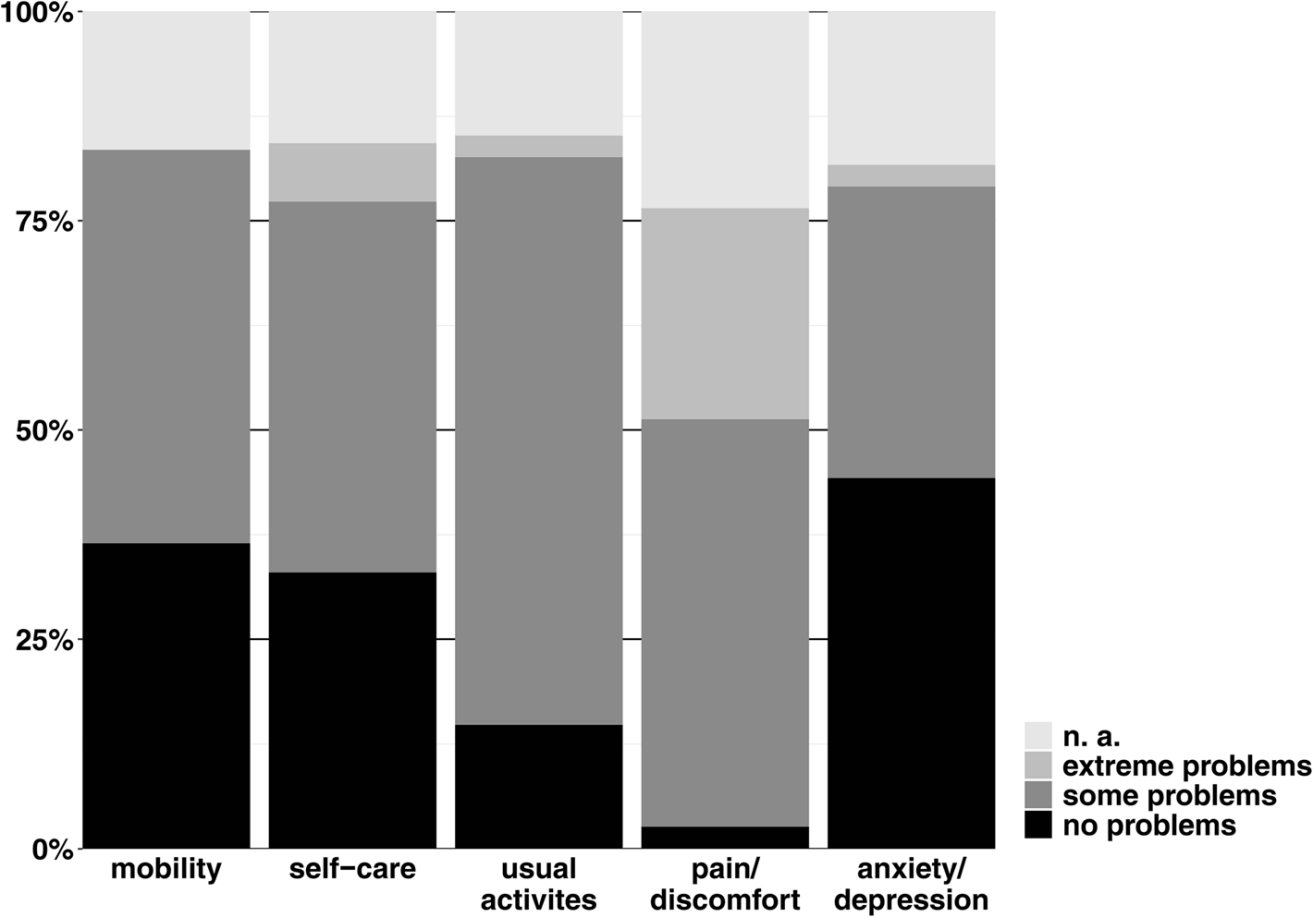
Dimensions of EQ-5D, P2015

Figure 3 shows the results of the descriptive system of the EQ-5D found in P2015, where 47.0% of the woman felt they had some problems with their mobility. Only 33.0% stated that they had no problems with their self-care, while 70.4% stated that they have “some problems” or are “unable performing their usual activities”. Only 2.6% reported no pain/discomfort and a mere 10.4% reported that they do not at all feel pain preventing them from doing what they need to do (in comparison to 26.2% in N2002 (see above and Fig. 3), *p* < .001). 37.4% reported that they feel sometimes or often anxious or depressed.

In P2015 of the 109 Thalidomide-impaired women who agreed to a psychiatric evaluation, 51 (46.8%) were diagnosed with some kind of current mental disorder (4 week prevalence). Of those 18 were facing a major depression and in 17 out of 18 cases the acute somatoform disorders found were pain disorders [34].

### Medical care and assistance

In N2002, 43.3% stated they had difficulties finding health professionals who understood their needs. 46.1% had been under medical treatment due to their impairment in the year before the survey.

In P2015 no problems with medical care were reported by 29.5% of the women, some problems by 57.6% and 8.1% faced extreme problems with their health care. There were 21 non-responders to that item. 48 (41.7%) women stated that they felt an additional appointment at a physician would have been necessary. The main reasons they did not see a physician were unavailability of a qualified physician (17.7%), total time of the appointment was too excessive (18.3%) and the feeling that the physician would have been unable to help (12.2%). Two women reported no problems with subsequent damage of their impairment by Thalidomide, 64 reported “some problems” with subsequent damages and 23 reported “extreme problems” with it. To this item were 26 non-responders.

Table 2 shows the involvement in assistance and/or nursing of the women affected by Thalidomide. Multiple answers were possible in this item, therefore subsets are not disjoint. The category “assistants/others” (see Tab. 3) includes professional as well as non-professional assistants since in the N2002 survey no distinction in this characteristic was made.

**Table 2:** Involvement in nursing/care of Thalidomide-impaired individuals, multiple answers were possible

	N2002 (*n* = 104)		P2015 (*n* = 115)		*p*-value
	n	%	n	%	
Spouse	48	46.1	44	38.3	.273
Parents	48	46.1	19	16.5	< .001^*^
Friends/Neighbors	48	46.1	28	24.3	.001^*^
Children	18	17.3	17	14.8	.713
Siblings	30	28.8	17	14.8	.014^*^
Ambulant services	6	5.8	10	8.7	.447
Assistants/Other	33	31.7	23	20.0	.062

^*^tested significantly at .05 level

In the N2002 sample 81.1% stated that they need assistance on a regular basis. With 5.8% community or private ambulant services were hardly utilized, while care was mostly provided by family members (63.6%) and friends (41.6%). In this survey was only asked for assistance, while in the P2015 questionnaire was asked for assistance as well as nursing. In the P2015 sample in 66 (57.4%) cases any kind of relatives or friends were involved in nursing/care. In 53.0% of all cases relatives (spouse, parents, siblings and children) were involved in nursing/care. Only in 12.2% of the cases the individuals made use of professional care or nursing. This includes ambulant services and five professional assistants.

The involvement of parents, siblings, friends and others decreased significantly, while the utilization of ambulant services did not rise in a corresponding manner (see Table 2). The prevalence for nursing found in P2015 exceeded the prevalence in the female German population of that age (about 8 ‰) by far (*p* < .001) [50].

## Discussion

The purpose of this publication was to describe the longitudinal effects of Thalidomide embryopathy and the subsequent impairments it causes on the life of women. Therefore the data of two cross-sectional studies (N2002 and P2015) were compared. Key findings included a declined working capacity, more prevalent health issues and lower satisfaction with health status in P2015 compared to the N2002 sample.

In the 12 years between the two studies the working capacity seems to have declined as many of the women in P2015 worked reduced hours and the proportion of women who were retired went up from 8.7% in the N2002 sample to 29.6% due to poor health in the P2015 sample. A small portion of decline in working hours might have been due to the –in some cases large– rise of the pension issued by the Contergan Foundation for Disabled People starting on January 1, 2013 [51], allowing individuals who had problems performing their workload to retire. The impact of the rise was extremely important but should also not be overestimated as the sole solution to the problem. For example a lack of knowledge by the medical personnel treating the impaired cannot be removed by providing financial aid to the impaired.

Health problems seem to become more prevalent and severe as the women age. This finding may be demonstrated by lower satisfaction with health status ratings and the statement of deteriorating health in the last 12 months. A majority of the women also reported the expectation of further deterioration in health. In addition somatoform disorders were found significantly more often than in the female general population of that age group in Germany [35, 52].

Nursing and/or care was assessed with different wording in the surveys. N2002 asked only for assistance while P2015 asked for assistance and/or nursing. Part of the decline in nursing and care might be due to this different approach in the two surveys. As the Thalidomide-impaired individuals grow older more assistance and/or nursing is needed. It can be anticipated that the assistance/nursing provided by non-professionals will likely become an issue since the greater part of the services are provided by the individuals’ parents and other non-professionals coming from the social environment of the impaired individuals. In the last 12 years the proportion of parents and other family members giving care has declined. But as the utilization of professional services has not risen to the same extent, it is likely that a concentration of care or nursing supplied by fewer non-professional caregivers with an extended workload for each of them has occurred. This will become an increasingly urgent matter due to the aging and therefore deteriorating health of the individuals’ informal caregivers. A gap in assistance and/or nursing will open up that sooner or later has to be filled by some kind of professional nursing and care. Especially for those women who are being cared for by their parents, who are estimated to be in their (late) seventies by now, this will become problematic, as the shortfall of one of those persons will lead to a severe problem: the women affected would encounter a healthcare system that is not prepared to satisfy their needs partially due to lack of knowledge.

### Comparison with other studies

To the authors’ best knowledge this publication compares for the first time the health status at two points in time in Thalidomide-impaired women.

### Strength and limitations of this study

One major strength of this comparison is the same sampling approach that was chosen in both studies with the same patient organization recruiting the participants. Most likely at least some of the women who participated in the survey of N2002 also participated in the study of P2015. Due to the differences found in the educational background it cannot be determined exactly how congruent the two samples were. The sample of P2015 might be biased in terms of education since the incentive of a full medical report and therapeutic suggestions on an individual basis were offered for participating in the study. Education has been shown in several studies to be associated positively to health related behavior [53, 54].

In a recent survey done 2013 by Kruse et al. [28] all over Germany (*n* = 870), the educational background in Thalidomide-impaired individuals was found to be of higher quality than the of the general German population at that age. The completion of a university degree among the age-adjusted females of the German general population is 8.6%, while it is significantly higher (*p* < .001) with 35.7% (see Table 1) in the results found in P2015.

Accordingly to the findings of N2002, Kruse found a significantly lower global Quality of Life than in the age-adjusted general German population.

Even with those deviations towards the same direction in several sociodemographic domains and the Quality of life found in Kruse, N2002 and P2015 it remains unknown how representative the samples were for all women affected by Thalidomide living in North Rhine-Westphalia. The self-selection bias for the willingness to take part in a study remains unknown for women in North Rhine-Westphalia. Both the samples of N2002 and P2015 seem not to be biased compared to Kruse [47] due to the sampling procedure via the same patient support group.

A further limitation is the presented comparison of self-reported data collected in N2002 with diagnoses found by a physician in P2015. The problems of self-reported diagnoses and their validity have been widely discussed [55–60]. Also problematic are the different instruments used to assess measures of health outcomes, of which some fit poorly together for comparisons.

As this is the best data available at this point in time for the specific population investigated, these limitations had to be accepted to allow a comparison over time. Due to the challenges described above it is highly desirable to have a longitudinal follow up on P2015. That study should use the same sample and study instruments, since it would give the possibility to assess a large sample of individuals with this orphan disease in a long-term setting and therefore describe the ongoing impairments caused by Thalidomide and their effects on the persons affected across their lifespan.

A first change has been made as the pensions received by the impaired have been risen in 2013 to a maximum of six times the previous highest pension [51]. Therefore at least some of the financial issues that the women feared would occur over time in their lives should be solved.

## Conclusion

The aim of this comparison of two cross-sectional studies about the life circumstances of women impaired by Thalidomide was to determine to what extent their situation has changed over the last 12 years. Even though the data was not always comparable, it is the best long term data available and therefore is the best possible assessment as of yet.

The results indicate an ongoing decrease in the status of health and the quality of life while their working capacity has declined due to health related problems and the legal disability to work has significantly risen. The prevalence of mental disorders of the women impaired by Thalidomide is increased compared to the female age-adjusted general German population [34]. Assistance and/or nursing for them is provided mostly by non-professional care-givers and in many cases by their parents which will become an issue as the established social networks fall apart with the aging of the care-givers and their need to be replaced. Since a lot of women do not feel that they get the full healthcare provided they need due to ignorance of their impairment and their medical needs by the medical professionals providing for them the following strategies were developed:

(i) to identify endangered individuals and offer tailored preventions and treatments

(ii) to minimize unmet medical needs and provide a supply scheme

(iii) to train the medical personnel providing health care for the impaired.

To truly assess the life conditions of the individuals impaired by Thalidomide over a period of time a long term study with a representative sample including both sexes and in a less restricted area of residence seems appropriate.

## Abbreviations

N2002: Survey done by Nippert et al. [30] in 2002
P2015: Study done by Peters et al. [29] in 2015

## References

[1] Lenz W (1988). A short history of thalidomide embryopathy. Teratology.

[2] Lenz W, Pfeiffer R, Kosenow W (1962). Thalidomide and congenital abnormalities. Lancet.

[3] Zuckerplätzchen forte. Der Spiegel. 1961:59–60.

[4] Gefahr im Verzuge. Der Spiegel. 1962:72–90.

[5] Vargesson N (2009). Thalidomide-induced limb defects: resolving a 50-year-old puzzle. Bioessays.

[6] Der KB (1999). Contergan-Fall: eine unvermeidbare Arzneimittelkatastrophe? Zur Geschichte des Arzneistoffes Thalidomid.

[7] Vargesson N (2013). Thalidomide Embryopathy: an enigmatic challenge. ISRN Developmental Biology.

[8] Miller MT, Strömland K (1999). Teratogen update: thalidomide: a review, with a focus on ocular findings and new potential uses. Teratology.

[9] Niethard FU, Marquard E, Eltze J (1994). Contergan: 30 Jahre danach.

[10] Strömland K, Nordin V, Miller M (1994). Autism in thalidomide embryopathy: a population study. Dev Med Child Neurol.

[11] Mcbride WG (1961). Thalidomide and congenital abnormalities. Lancet.

[12] Lenz W, Knapp K (1962). Thalidomide embryopathy. Dtsch Med Wochenschr.

[13] Withdrawal of Thalidomide from the Market. Can Med Assoc J 1962;**86**(14):664. PMC1849348 20327092

[14] Kajii T, Shinohara M (1963). Thalidomide in Japan. Lancet.

[15] Ito T, Ando H, Suzuki T (2010). Identification of a primary target of thalidomide teratogenicity. Science.

[16] Knobloch J, Rüther U (2008). Shedding light on an old mystery: thalidomide suppresses survival pathways to induce limb defects. Cell Cycle.

[17] Kim JH, Scialli AR (2011). Thalidomide: the tragedy of birth defects and the effective treatment of disease. Toxicol Sci.

[18] Castilla EE, Ashton-Prolla P, Barreda-Mejia E (1996). Thalidomide, a current teratogen in South America. Teratology.

[19] Singhal S, Mehta J (2002). Thalidomide in cancer. Biomed Pharmacother.

[20] Licht JD, Shortt J, Johnstone R (2014). From anecdote to targeted therapy: the curious case of thalidomide in multiple myeloma. Cancer Cell.

[21] Singhal S, Mehta J, Desikan R (1999). Antitumor activity of thalidomide in refractory multiple myeloma. N Engl J Med.

[22] Lazzerini M, Martelossi S, Magazzù G (2013). Effect of thalidomide on clinical remission in children and adolescents with refractory Crohn disease: a randomized clinical trial. JAMA.

[23] Stephens T, Brynner R (2001). Dark remedy: The impact of thalidomide and its revival as a vital medicine.

[24] Marwick C (1997). Thalidomide back--under strict control. JAMA.

[25] Zeldis JB, Williams BA, Thomas SD (1999). S.T.E.P.S.: a comprehensive program for controlling and monitoring access to thalidomide. Clin Ther.

[26] Rocha J (1994). Thalidomide given to women in Brazil. BMJ.

[27] Paumgartten FJR, Chahoud I (2006). Thalidomide embryopathy cases in Brazil after 1965. Reprod Toxicol.

[28] Kruse A, Ding-Greiner C, Becker G (2012). CONTERGAN Wiederholt durchzuführende Befragung zu Problemen, speziellen Bedarfen und Versorgungsdefiziten von contergangeschädigten Menschen.

[29] Peters KM, Albus C, Lüngen M (2015). Gesundheitsschäden, psychosoziale Beeinträchtigungen und Versorgungsbedarf von contergangeschädigten Menschen aus Nordrhein-Westfalen in der Langzeitperspektive: Forschungsbericht. Studie im Auftrag des Landeszentrums Gesundheit Nordrhein-Westfalen.

[30] Nippert I, Edler B, Schmidt-Herterich C (2002). 40 years later: the health related quality of life of women affected by thalidomide. Community Genet.

[31] Wittchen HU, Zaudig M, Fydrich T (1997). Strukturiertes klinisches Interview für DSM-IV: Achse I und II.

[32] Spitzer RL, Williams JB, Gibbon M (1992). The structured clinical interview for DSM-III-R (SCID). I: history, rationale, and description. Arch Gen Psychiatry.

[33] Spitzer RL, Williams J, Gibbon M (1990). First, M.B. Structured clinical interview for DSM-III-R.

[34] First MB, Gibbon M, Spitzer RL (1997). Structured clinical interview for DSM-IV Axis II personality disorders, (SCID-II).

[35] Niecke A, Peters K, Samel C (2017). Mental disorders in people affected by thalidomide. Deutsches Arzteblatt Int.

[36] The WHOQOL Group (1998). Development of the World Health Organization WHOQOL-BREF quality of life assessment. The WHOQOL group. Psychol Med.

[37] Angermeyer MC, Kilian R, Matschinger H (2000). WHOQOL-100 und WHOQOL-BREF: Handbuch für die deutschsprachige Version der WHO Instrumente zur ERfassung von Lebensqualität.

[38] Morfeld M, Kirchberger I, Bullinger M (2011). SF-36 Fragebogen zum Gesundheitszustand: Deutsche Version des Short Form-36 Health Survery.

[39] Ware JE, Sherbourne CD (1992). The MOS 36-item short-form health survey (SF-36). I. Conceptual framework and item selection. Med Care.

[40] van Reenen M, Oppe M. EQ-5D-3L User Guide: Basic information on how to use the EQ-5D-3L instrument. Version 5.1. 2015. Available at: https://euroqol.org/wp-content/uploads/2016/09/EQ-5D-3L_UserGuide_2015.pdf Accessed 25 Mar 2019.

[41] The EuroQol Group (1990). EuroQol--a new facility for the measurement of health-related quality of life. Health Policy.

[42] Brooks R (1996). EuroQol: the current state of play. Health Policy.

[43] Bundesministerium der Finanzen. Umrechung DM/Euro 2001. Available at: https://www.bundesfinanzministerium.de/Content/DE/Downloads/Europa/uebersicht-euro-umrechnung.pdf;jsessionid=D7C427E18B61286B8B78140A3231ED16?__blob=publicationFile&v=3. Accessed 25 Mar 2019.

[44] Statistisches Bundesamt. Verbraucherpreisindizes: Verbraucherpreise 2015. Available at: https://www.destatis.de/GPStatistik/servlets/MCRFileNodeServlet/DEHeft_derivate_00039689/Jahresbericht_2015.pdf Accessed 26 Mar 2019.

[45] Deutsche Rentenversicherung Bund. Rentenbestand am 31.12.2013: Statistik der Deutschen Rentenversicherung 2014. Available at: www.fdz-rv.de/FdzPortalWeb/resDisplay.do?id=2185&tabelle=3. Accessed 25 Mar 2019.

[46] Statistisches Bundesamt. Daten zu Geburten, Familien und Kinderlosigkeit: Ergebnisse des Mikrozensus 2012, Tabellen mit neuer Hochrechnung anhand der Bevölkerungsfortschreibung auf Basis des Zensus 2011. 2015. Available at: https://www.destatis.de/GPStatistik/servlets/MCRFileNodeServlet/DEMonografie_derivate_00001863/5122203159014.pdf. Accessed 26 Mar 2019.

[47] Statistisches Bundesamt. Alleinlebende in Deutschland: Ergebnisse des Mikrozensus 2011. Ergänzende Tabellen zur Pressekonferenz am 11.Juli 2012 in Berlin 2012. Available at: https://www.destatis.de/GPStatistik/servlets/MCRFileNodeServlet/DEMonografie_derivate_00001441/Alleinlebende.pdf. Accessed 26 Mar 2019.

[48] Statistisches Bundesamt. Einkommen, Einnahmen & Ausgaben: Einkommen, Einnahmen und Ausgaben deutscher Haushalte im Zeitvergleich. Available at: https://www.destatis.de/DE/Themen/Gesellschaft-Umwelt/Einkommen-Konsum-Lebensbedingungen/Einkommen-Einnahmen-Ausgaben/Tabellen/liste-deutschland.html. Accessed 26 Mar 2019.

[49] Bundesministerium für Familie, Senioren, Frauen und Jugend. Conterganstiftungsgesetz 2013. Available at: https://www.bmfsfj.de/bmfsfj/aktuelles/alle-meldungen/conterganstiftungsgesetz/77546. Accessed 25 Mar 2019.

[50] Statistisches Bundesamt. Pflegestatistik 2013: Pflege im Rahmen der Pflegeversicherung, Deutschlandergebnisse. 2015 Available at: https://www.destatis.de/GPStatistik/servlets/MCRFileNodeServlet/DEHeft_derivate_00015401/5224001139004.pdf. Accessed 26 Mar 2019.

[51] Bundesministerium für Familie, Senioren, Frauen und Jugend. Übersicht: Höhe der Leistungen nach Schadensstufen 2013. Available at: https://www.bmfsfj.de/blob/78340/054f85ec853b559757308c8586a5ca66/conterganstiftung-gesetzentwurf-data.pdf. Accessed 25 Mar 2019.

[52] Jacobi F, Höfler M, Siegert J (2014). Twelve-month prevalence, comorbidity and correlates of mental disorders in Germany: the mental health module of the German health interview and examination survey for adults (DEGS1-MH). Int J Methods Psychiatr Res.

[53] Davey Smith G, Hart C, Hole D (1998). Education and occupational social class: which is the more important indicator of mortality risk?. J Epidemiol Community Health.

[54] Lantz PM, House JS, Lepkowski JM (1998). Socioeconomic factors, health behaviors, and mortality. JAMA.

[55] Schneider ALC, Pankow JS, Heiss G (2012). Validity and reliability of self-reported diabetes in the atherosclerosis risk in communities study. Am J Epidemiol.

[56] Navarro C, Chirlaque MD, Tormo MJ (2006). Validity of self reported diagnoses of cancer in a major Spanish prospective cohort study. J Epidemiol Community Health.

[57] Meltzer JW, Hochstim JR (1970). Reliability and validity of survey data on physical health. Public Health Rep.

[58] Rasooly I, Papageorgiou AC, Badley EM (1995). Comparison of clinical and self reported diagnosis for rheumatology outpatients. Ann Rheum Dis.

[59] Warren MD (1976). Interview surveys of handicapped people: the accuracy of statements about the underlying medical conditions. Rheumatol Rehabil.

[60] Schrag A, Brown RJ, Trimble MR (2004). Reliability of self-reported diagnoses in patients with neurologically unexplained symptoms. J Neurol Neurosurg Psychiatry.

